# Sample-based approach can outperform the classical dynamical analysis - experimental confirmation of the basin stability method

**DOI:** 10.1038/s41598-017-05015-7

**Published:** 2017-07-21

**Authors:** P. Brzeski, J. Wojewoda, T. Kapitaniak, J. Kurths, P. Perlikowski

**Affiliations:** 1 Division of Dynamics, Lodz University of Technology, 90-924 Lodz, Poland; 2 Potsdam Institute for Climate Impact Research, Potsdam, 14415 Germany; 3 Institute of Physics, Humboldt University of Berlin, Berlin, 12489 Germany

## Abstract

In this paper we show the first broad experimental confirmation of the basin stability approach. The basin stability is one of the sample-based approach methods for analysis of the complex, multidimensional dynamical systems. We show that investigated method is a reliable tool for the analysis of dynamical systems and we prove that it has a significant advantages which make it appropriate for many applications in which classical analysis methods are difficult to apply. We study theoretically and experimentally the dynamics of a forced double pendulum. We examine the ranges of stability for nine different solutions of the system in a two parameter space, namely the amplitude and the frequency of excitation. We apply the path-following and the extended basin stability methods (Brzeski *et al*., Meccanica 51(11), 2016) and we verify obtained theoretical results in experimental investigations. Comparison of the presented results show that the sample-based approach offers comparable precision to the classical method of analysis. However, it is much simpler to apply and can be used despite the type of dynamical system and its dimensions. Moreover, the sample-based approach has some unique advantages and can be applied without the precise knowledge of parameter values.

## Introduction

There is a rich variety of different mathematical tools to analyze nonlinear dynamical systems. Still, more sophisticated methods are usually difficult to apply. For example, there are a number of different toolboxes that enable the path-following analysis but their functionality is strictly limited to the type of the investigated system and its dimensionality. The dynamical analysis is especially challenging for multistable systems, where we have to consider multiple steady states that coexist in the phase space. It is a challenging problem and multistability is widely studied in many disciplines^1–8^. Therefore, also new tools to analyze multistable systems are being developed.

In 2013 Menck *et al*. proposed a basin stability measure that uses Bernoulli trials to estimate the volume of a basin of attraction^9^. Despite it is new, the method was already successfully applied in numerous different applications^10–13^. In 2016 another new tools to investigate multistable systems were proposed, namely survivability^14^ that includes the analysis of the transient motion and basin entropy^15^ that measures the basin compactness.

The growing interest in sample-based methods comes from two main advantages. They can be easily applied to all types of systems and reproduce the inherent uncertainty of perturbations.british To apply these methods, we just need to have a reliable direct numerical integration code for the mathematical model. Also the computational effort does not grow significantly with the increase of the phase space dimensions. This makes such sample-based methods even more appealing for the analysis of very high-dimensional systems such as large networks of oscillators, power grids^16^ or brain dynamics^17^. However, up to this moment there is a lack of experimental studies which enable to compare the accuracy of these methods and classical analysis.

Recently^18^ we described an extended basin stability approach by taking into account mismatch in parameter values. This has practical fundamentals because all parameters values are measured or estimated with some finite precision and can slightly vary even during normal operation. In this paper, we expand our approach and perform an analysis in a two parameter space. We compare the results from sample-based analysis with detailed two parameter bifurcation diagrams obtained using the path-following method. Finally, we confront both methods with experimental data that we use as a benchmark. This is done for 9 different periodic solutions that coexist in a notably wide range of the parameter values. The results enable us to critically compare the accuracy of both methods and show their strengths and weaknesses. Apart from that, we show that the sample-based approach can be applied without a precise knowledge of parameter values and it gives sensible results.

## Model of system

**Figure 1:**
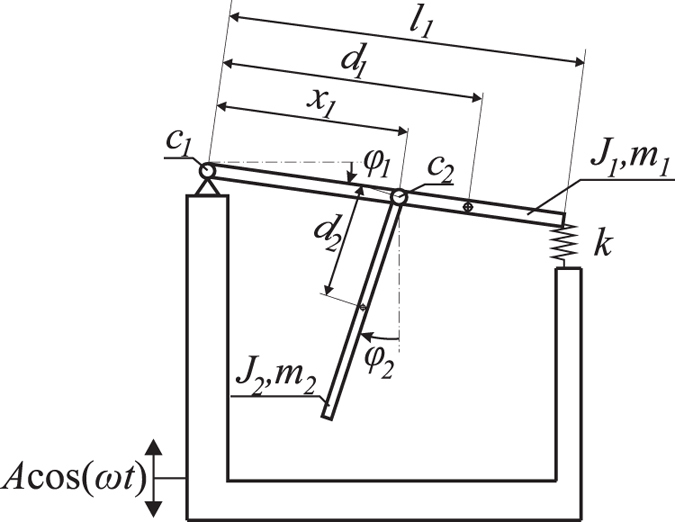
The physical model of the considered double pendulum excited kinematically (Eq. 1) with its parameters.

We consider the specific type of a double pendulum (see Fig. 1) which is a paradigmatic example in nonlinear dynamics. The first pendulum rod is mounted horizontally and connected to the base with a pin joint at one end and via a spring on the second end. Hence, it can only oscillate. The second pendulum is connected to the first one with a rotational pivot at the distance *x*_1_ between both pin joints. The support is mounted on a shaker and excited kinematically in the vertical direction. This system is a modified version of the rig considered by Strzalko *et al*.^19^ and Dudkowski *et al*.^1^.

The angular displacements of the first and the second pendulum are given by *φ*_1_ and *φ*_2_ respectively. The shaker excites the system kinematically with a harmonic function of the amplitude *A* and frequency *ω*. The upper pendulum has the length *l*_1_, the mass *m*_1_, the moment of inertia *J*_1_ and the centre of gravity located at the distance *d*_1_ from its pin joint. The stiffness of the spring that supports the end of the pendulum is given by *k*. The second pendulum has the mass *m*_2_, the moment of inertia *J*_2_ and the centre of gravity located at *d*_2_ from its pin joint. Due to the relatively small damping in the system we neglect effects of dry friction and other non-linearities of damping characteristics and in both pivots we assume the viscoelastic damping. Hence, for the first pendulum the damping coefficient is given by *c*_1_ and for the second one by *c*_2_.

The equations of motion of the system shown in Fig. 1 are given by the following second order ODEs:

1 $$\begin{array}{l}({J}_{2}+{m}_{2}{d}_{2}^{2}){\ddot{\phi }}_{2}+{m}_{2}{d}_{2}(A{\omega }^{2}\,\cos \,(\omega t)+g)\,\sin \,{\phi }_{2}\\ \begin{array}{ccc}\quad +{m}_{2}{d}_{2}({x}_{1}(\cos \,({\phi }_{1}-{\phi }_{2})\,{\dot{\phi }}_{1}^{2}+\,\sin \,({\phi }_{1}-{\phi }_{2}){\ddot{\phi }}_{1}))+{c}_{2}{\dot{\phi }}_{2} & = & 0,\end{array}\\ ({J}_{1}+{m}_{1}{d}_{1}^{2}+{m}_{2}{x}_{1}^{2}){\ddot{\phi }}_{1}+{c}_{1}{\dot{\phi }}_{1}+\frac{1}{2}{l}_{1}^{2}k\,\sin \,(2{\phi }_{1})\\ \quad +{m}_{2}{x}_{1}{d}_{2}(\sin \,({\phi }_{1}-{\phi }_{2}){\ddot{\phi }}_{2}-\,\cos \,({\phi }_{1}-{\phi }_{2}){\dot{\phi }}_{2}^{2})+\\ \begin{array}{ccc}\quad \quad \quad \quad \quad \quad \quad \,-({m}_{1}{d}_{1}+{m}_{2}{x}_{1})(A{\omega }^{2}\,\cos \,(\omega t)+g)\,\cos \,{\phi }_{1} & = & 0.\end{array}\end{array}$$

The parameters have the following values: *J*_1_ = 4.524[10^−3^ kgm^2^], *m*_1_ = 0.5562 [kg], *l*_1_ = 0.315 [*m*], *d*_1_ = 0.180 [m], *x*_1_ = 0.153[m], *c*_1_ = 0.05 [Nms], $k=6850\,[\underline{{\rm{N}}}]$, *J*_2_ = 4.469 [10^−5^ kgm^2^], *m*_2_ = 0.02077[kg], *d*_2_ = 0.063[m], *c*_1_ = 7[10^−6^ Nms], *g* = 9.81[(m)/(s^2^)]. All the parameter values were determined in a series of dedicated experiments.

## Methods

We compare the results obtained by using two different approaches and validate them with experiments. As aforementioned, the considered system is a double pendulum forced kinematically in vertical direction (Eq. 1). As controlling parameters we take the amplitude *A* and the frequency *ω* of the external excitation. The system is multistable, hence we observe a coexistence of solutions for fixed parameter values. Different solutions stabilize and destabilize when varying the amplitude and/or frequency of excitation. The aim of our study is to investigate the ranges of attractors’ stability in the (*A*, *ω*) plane. We start with the path-following method to get a precise boundaries of solutions’ stability. Then, for each solution we perform experiments. Finally, we apply the sample-based approach (the extended basin stability method^18^) to determine the ranges of parameters for which the given solution can be obtained. In the following subsections, we describe the applied methods and explain the scheme in which we present the results. This is crucial because we present and compare a large amount of results obtained with various methods. Due to limitations of the experimental rig, the maximum value of the amplitude of excitation is *A*_*max*_ = 7.7[10^−3^ *m*] (higher amplitudes of the shaker are impossible to achieve). Similarly, we consider the frequency of excitation in the range *ωε*〈0, 60〉[*rad*/*s*], because for higher values the accessible amplitude of the excitation *A*_*max*_ is rapidly dropping.

### Path-following analysis

In numerical study we consider the following ranges of the amplitude *Aε*〈0, 7.7〉[10^−3^ *m*] and the frequency of excitation *ωε*〈0, 60〉[*rad*/*s*]. We perform path-following analysis for 9 different solutions. The upper pendulum always performs an oscillatory motion, while for the second pendulum we observe both oscillations and rotations with different locking ratios in respect to the frequency of the excitation. Therefore, we name each solution basing on the behaviour of the second pendulum and ratio *n*: *m* which means that for *m* periods of excitation we observe *n* full oscillations or rotations of the second pendulum. We detected and further consider: 1:1, 1:2, 1:3, 1:4, 1:6, 1:8 oscillations, and 1:1, 1:2, 1:3 rotations.

**Figure 2:**
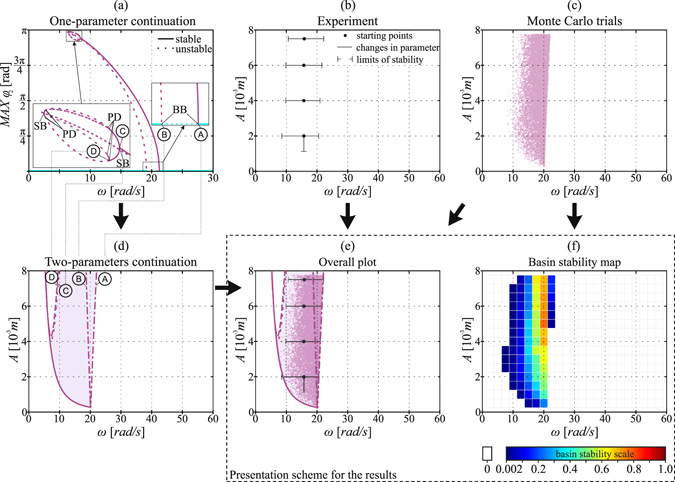
Presentation scheme for the stability analysis obtained using different approaches for 1:2 oscillatory solution. Panels (a,d) were obtained from the continuation of a periodic solution, in panel (b) we present experimental data and in panels (c,f) the results obtained with a sample-based method. In subplot (**c**) we compare the three approaches. Abbreviation used in panel (a) indicate the following bifurcations: PD - period doubling, SB - symmetry breaking pitchfork and BB - branching point where the equilibrium destabilizes and lower pendulum starts to move. Points where the bifurcations occur in panels (a) and (d) are marked with capital letters A–D. Henceforth, we will use two figures such as panels (e) and (f) to provide critical comparison between the methods.

For all 9 considered solutions we perform the path-following analysis by using the AUTO-07*p* software^20^. Each time we start with a direct numerical integration to prepare the initial periodic solution for continuation. Then, we start with one parameter continuation using *A* or *ω* as a continuation parameter. We detect the boundaries of stability and perform continuation of the bifurcation points in two parameter space. In Fig. 2(a,d,e) we present the sample results obtained this way (for 1:2 oscillations). For each considered solution we use different colour of lines and differentiate the line type for different types of bifurcations. For saddle-node (SN) bifurcations we use continuous lines, for branching bifurcations (BB) dash-dotted, for symmetry breaking (SB) dotted and for period doubling (PD) dashed lines. In the investigated system (Eq. 1) we often encounter a period doubling cascade. In such cases we indicate only the first and the second period doubling bifurcations.

### Experimental investigation

For the solutions detected numerically, we experimentally find the boundaries of the stability in the two parameter space (*A*, *ω*). The starting points for the experimental study are located far from the boundary of stability to ensure that the given solution is reachable experimentally. Depending on the shape and area of the stability range, we select from one to seven starting points and for each apply the following procedure:

- We start the forcing with the amplitude and the frequency of the excitation taken from numerical results and try to reach the given solution by applying proper initial conditions to the second pendulum. The knowledge of the time trace of the solution from AUTO-07*p* software helps us to apply the correct initial state.
- When the system reaches the presumed state we mark the amplitude and the frequency of excitation. These are the coordinates of a starting point in the two parameter space. Such points are marked with black dots in Fig. 2(b,e).
- Then, we slowly change the value of the frequency or the amplitude of forcing with small steps (minimal step in frequency is 2*π* × 10^−3^[*rad*/*s*] and in amplitude 0.01[*m*]). After each step we wait to check if the system remains on the presumed attractor. The range of the parameter value where the solution stays stable is marked as a trace with a black line in Fig. 2(b,e).
- Eventually, we reach the parameter value for which the solution becomes unstable and the system jumps to a different attractor. We note the amplitude and the frequency of excitation for which the solution changed.
- We repeat the above steps four times and compute the average values of parameters for which the solution looses its stability. We take this value as the boundary of stability and mark it with the perpendicular end of the stability trace (see Fig. 2(b,e).

To estimate the position of a line that indicates the boundary of stability obtained by the path-following method in the two parameter space (*A*, *ω*), we repeat the above procedure for different starting points. We detect the ranges of stability for all 9 encountered solutions which required enormous effort and a lot of time.

### Extended basin stability approach

Now we apply the extended basin stability approach described in our recent work^18^. To start with, we have to estimate the ranges of initial conditions that can be applied on our experimental rig. In a number of dedicated experiments we defined the accessible ranges of initial conditions that describe physical restrictions of our rig: *φ*_1_ *∈*〈−0.0015,0.0015〉[*rad*], *φ*_2_ *∈*〈−*π*, *π*〉[*rad*], ${\dot{\phi }}_{1}{\in }\langle -\mathrm{1.2,}\,1.2\rangle \,[rad/s]$, ${\dot{\phi }}_{2}{\in }\langle -\mathrm{60,}\,60\rangle \,[rad/s]$. Then, we divide the considered ranges of parameter values into a regular two dimensional grid in the following way: for the frequency of excitation (*ω∈*〈0, 60〉[*rad*/*s*]) we assume 23 subsets with equal width of 25[*rad*/*s*]. We intentionally omit extremely small values of *ω* and start from the range 〈1.25,3.75〉[*rad*/*s*] (for first column of the grid) and finish with 〈56.25, 58.75〉[*rad*/*s*] (for the last column). For the amplitude of forcing *A∈*〈0, 7.7〉[10^−3^ *m*] we take 15 equally spaced subsets with the step of 0.5 [10^−3^ *m*]. Here, we also ignore values near the accessible boundaries and start the lower row of the grid with the range of 〈0.25, 0.75〉[10^−3^ *m*] and finish the grid with 〈7.25, 7.75〉[10^−3^ *m*]. By that, we receive a lattice of 345 boxes each covering the range of 2.5 [*rad*/*s*] and 0.5 [10^−3^ *m*] of the forcing frequencies and amplitudes respectively.

For each box in the grid we perform 500 trials of direct numerical integration. Each time we randomly pick the initial conditions from the accessible ranges and draw the values of *A* and *ω* from the ranges assigned to the grid box. For every trial we recognize the final attractor that is reached by the system. In a procedure described above, we obtain a large data set of 172, 500 Monte Carlo trials that we use to characterize the possible behaviours of the system.

The data enable us to estimate the ranges of stability for each considered attractor. For that purpose, we detect all the trajectories that go to the investigated attractor and mark the parameters value (*A* and *ω*) for which it has been approached. Then, we plot those values as a set of points in the (*A*, *ω*) plane that indicates the region of stability of the investigated solution. In Fig. 2(c) we present the outcome of such analysis for 1:2 oscillations. Apart from that, the collected data enable us to calculate the maps of basin stability in the two parameter space, as shown in Fig. 2(f) where we show the probability of occurrence of the considered solution in each of 345 boxes.

### Presentation of the results

For every detected solution we get the boundaries of stability using the above methods. To show all of the obtained results and ensure an easy comparison between the methods, we need to develop a clever presentation scheme. In Fig. 2 we present the results yielded for 1:2 oscillations to explain the presentation scheme used throughout the paper. Arrows in Fig. 2 show how the data are interchanged between the panels.

We start with the path-following method. Panel (a) presents the one parameter continuation performed to detect the bifurcation points in which the stability of the solution changes. We perform two parameter continuation for these points, as shown in panel (d). We also mark the region of stability with a coloured area. In panel (b) we show the experimental results, while in panels (c) and (f) we present the outcome of the sample-based approach. We plot a single colour dot (here purple) each time the investigated solution is reached in a trial. The extended basin stability approach provides a basin stability map shown in panel (f). Such plot enables us to detect where the solution is more likely to appear due to the increased basin stability. Moreover, thanks to the lattice, we maintain a consistent level of error (smaller than 2.25%) because in each box we have 500 Bernoulli trials.

To simplify the comparison between the results obtained with different analysis approaches, we combine the results from panels (d), (b) and (c) in a single plot such as Fig. 2(e). However, we think that the maps of basin stability provide an additional knowledge about the attractor stability. Hence, for each solution we present two diagrams corresponding to panels (e) and (f). To underline this fact we mark these two panels with a dashed line rectangle.

## Results

The aim of our investigations is to compare different analysis approaches and experimentally validate the accuracy of the sample-based method. In this section we provide a thorough presentation of the results obtained using the path-following method, experimental investigations and the extended basin stability technique. We focus on the detection of the ranges of stability for 9 solutions mentioned in previous section. Moreover, we supplement the results with basin stability maps that enable us to quantify the stability and indicate the ranges of parameter values for which it is more likely that given solution occurs.

### Ranges of stability

**Figure 3:**
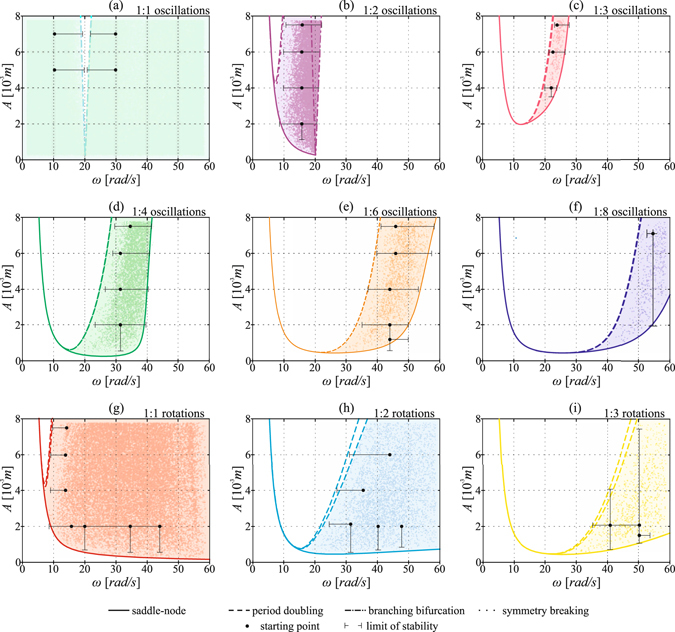
Comparison between the ranges of stability obtained using the path-following method (colour lines), the sample-based approach (dots) and the experimental investigation (black lines). Each panel correspond to a different solution. Colour line type depicts the type of bifurcation.

We investigate the boundaries of stability for 9 different solutions that coexist in a wide range of the parameter values. The results are presented in Fig. 3 using the presentation scheme described in subsection “Presentation of the results”.

In panel (a) of Fig. 3 we show the results obtained for 1:1 oscillations of the vertical pendulum with very small amplitude (typically this solution is reported as a semi-trivial one, because in the system with significantly larger damping the motion of the vertical pendulum would be not or barely observable). This solution is stable in the whole analysed range of the parameter values except the narrow resonance tongue that occurs for *ω* = 20[*rad*/*s*]. The position of the branching bifurcation lines have been detected by path-following and confirmed using both experimental analysis and sample-based approach. The agreement between the results obtained using all three approaches is remarkably good.

The second analysed solution is 1:2 oscillations which corresponds to the resonance tongue (around *ω* = 20[*rad*/*s*]). The results are shown in Fig. 3(b). The path following analysis reveals that this solution loses its stability either in a saddle node bifurcation (for *A* ≤ 5 [10^−3^ *m*]) or in a symmetry braking bifurcation (for *A* > 5 [10^−3^ *m*]) that is immediately followed by a cascade of period doubling bifurcations. The experimental analysis confirms that the position of the right-hand side border of the resonance tongue has been obtained with an excellent precision. However, the detected range of stability differs from the results obtained via the numerical continuation. The difference is especially visible for *A* = 4 [10^−3^ *m*]s (in horizontal direction) and for *ω* = 16[*rad*/*s*] in vertical direction. In these cases the sample-based method enables a better precision, especially when detecting the left-hand side border of stability. We see that the density of points decreases significantly before the bifurcation lines are reached. Thus using the sample-based approach we can better predict the range in which we are able to obtain 1:2 oscillations.

The third analysed solution (panel (c)) is 1:3 oscillations. Here, the results from both methods of analysis are in good agreement with the experimental data. Similarly, for 1:6 oscillations (panel (e)) and 1:8 oscillations (panel (f)) we observe a concurrence of the results from all the applied methods. The difference between the experimental and numerical results is clearly distinguishable only in two cases. The experimental investigation revealed that the 1:6 oscillations can be achieved also below the line detected using the path-following method. Also, the position of a period doubling bifurcation which destroys the stability of 1:8 oscillations is different from what we observe experimentally. We think that the difference between the numerical results and the experiment mainly comes from the dissipation modelling approach which strongly simplifies this phenomenon. In panel (d) we show the results that correspond to 1:4 oscillations. For this solution, with the Monte Carlo approach can obtain the boundary of stability that is closer to the experimental results then the bifurcation lines obtained via numerical continuation. It is especially visible for the left and the lower boundaries in the (*A*, *ω*) plane.

The last three solutions presented in panels (g,h,i) correspond to 1:1, 1:2 and 1:3 rotations respectively. For all rotary solutions we see a good convergence between the experiments and the results obtained using both numerical methods.

The results presented in Fig. 3 prove that for the considered system the path-following method and the sample-based approach offer similar accuracy when compared to experimental results. The important advantage of the sample-based approach is that during a large number of trials we are able to detect hidden attractors^21^ or solutions with rather meager basins of attraction. In the investigated case we also found solutions that occurred only once for 172, 500 trials, such as for example 2:5 oscillations or 3:15 rotations. Moreover, analysing Fig. 3, we see that with the sample-based approach we find the regions with the maximum density of points. This, in consequence enables to quantify the stability for different values of parameters using the basin stability measure. To further investigate this topic for each solution we obtain maps indicating the changes of basin stability in the two-parameter space.

### Basin stability maps

**Figure 4:**
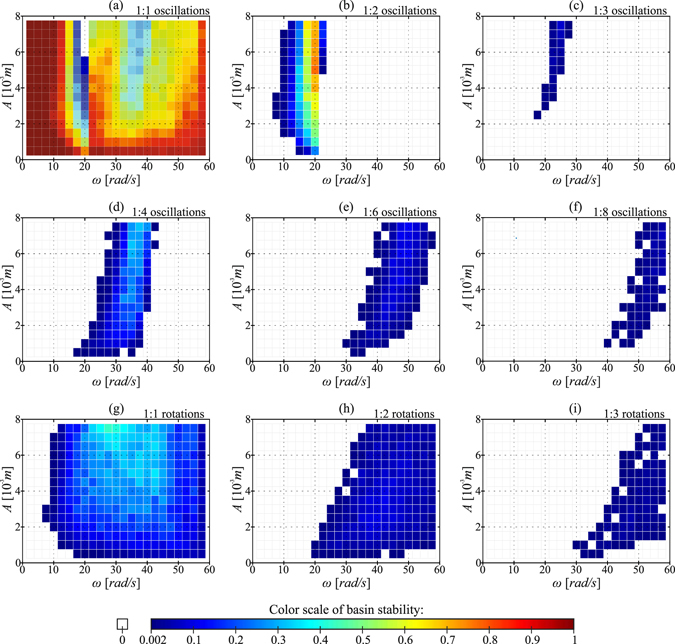
Two parameters maps of the basin stability for the 9 investigated solutions. The grid corresponds to the lattice used during the calculations. Below is the common basin stability scale used for all 9 panels.

The results presented in the previous subsection indicate that we are able to achieve comparable precision using the sample-based approach. Now, we show the additional information that can be obtained with extended basin stability method^18^. In Fig. 4 we demonstrate the changes of basin stability in the two parameter space for the 9 considered solutions. We present the results in the grid in which the calculations were performed. For all presented diagrams we hold the same colour scale for the basin stability that is given at the bottom of Fig. 4.

Panel (a) refers to the 1:1 oscillations which has the largest range of stability (see Fig. 2(a)). The plot reveals that also in the large range of parameters this solution has the dominant volume of the basin of attraction. Analysing the panels (b–f) which correspond to the oscillatory solutions, we find that for most of them basin stability calculated in the assumed grid never exceeds 30%. Still, for 1:2 and 1:4 oscillations we uncover regions with higher basin stability. For rotary solutions (panels (g–i)) we observe the similar tendency and the basin stability exceeds 15% only for 1:1 rotations.

Comparing all the diagrams in Fig. 3, we can divide the considered range of parameter values into three regions:

1. The range where the semi-trivial solution has the dominant basin of attraction (see panel (a)).
2. The resonance tongue of 1:2 oscillations in which this solution has the biggest volume of the basin (panel (b)).
3. The region where, we observe the coexistence of many stable solutions and the 1:1 rotations has the dominant basin of attraction in most of that region (panel (g)).

Using the data obtained in a large series of trials we can use an interpolation and draw the continuous maps of basin stability (not using the lattice). We chose to present the data in the grid in order to maintain a similar level of error and to indicate the methodology of our approach. Despite the presentation method, basin stability maps enable to detect where the given solution has the biggest relative volume of the basin of attraction. This in consequence, refers to the ranges in parameter space for which the solution is more likely to appear. Therefore, such diagrams have strong practical significance.

### Investigation of the system with parameters mismatch

In practical applications we often cannot obtain the exact values of the system’s parameters. It is especially difficult when the model contains parameters whose values cannot be measured directly such as, for example, a value of the viscous damping coefficient in a pin joint. Apart from that, when modelling the mechanical and structural objects, we often simplify complex phenomena using simple models. This can decrease the accuracy of simulations and cause the divergence between numerical and experimental results. In this section we show that the extended basin stability method can be applied without the knowledge of actual parameter values and still maintain the high accuracy of the yielded results.

Let us assume that we do not know precisely the values of some parameters of the system. In a classical approach, we have to set their values which may lead to wrong results. In the sample-based approach, instead of setting a value of the parameter, we can estimate the range to which this parameter belongs. Then, during a series of trials we draw the values of uncertain parameters. Before we apply that to our problem, we divide the parameters of our system (Eq. 1) into four specific groups basing on the ease of measurements:

1. Parameters that are easy to measure: *m*_1_, *m*_2_, *l*_1_, *x*_1_. This group contains the parameters that can be determined easily with good precision, namely masses and lengths of the physical objects. During calculations we assume that we determined values of these parameters precisely and do not draw them.
2. Parameters that can be estimated with good precision: *J*_1_, *J*_2_, *d*_1_, *d*_2_, *k*. The methods to measure these parameters values are not straight forward. In this group there are also parameters whose values are given by manufacturer with certain precision - as for example the stiffness of the spring. We assume that the error for the parameters from that group is ±5% and for each trial we draw the values of parameters from a certain range.
3. Parameters that are difficult to estimate: *c*_1_, *c*_2_. In this group we put parameters that require complex procedures or approach to infer their values and parameters that refers to phenomena that are difficult to model, such as energy dissipation. Values of parameters from this group are drawn from the range 〈−120%, 120%〉 of the estimated value.
4. Parameters whose special influence we investigate: *A*, *ω*. The aim of the study is to analyse the influence of these parameters on the stability of solutions. For these parameters we do not estimate the error. Instead, we draw values of these parameters from a “grid” as described in the Methods section.

Apart from the above, we want to maintain the value of the natural frequency of the second pendulum which can be easily measured using a simple stopwatch. For that purpose, we draw the inertia of the second pendulum and recalculate the position of its centre of gravity to preserve the natural frequency.

**Figure 5:**
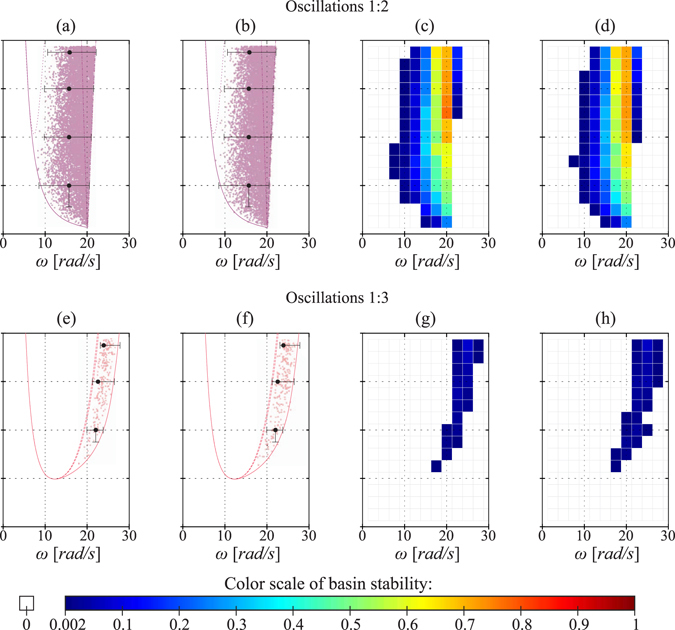
Comparison between the results obtained from the model with and without the parameters mismatch. Panels (a,c,e,g) presents the results from sample-based approach with fixed parameters while panels (b,d,f,h) with parameters mismatch (parameters are drawn from the assumed ranges).

With the above assumptions we repeat all the calculations. In Fig. 5 we present the comparison between the results obtained with fixed values of parameters and when drawing the parameter values. In the upper row of Fig. 5 we show the results that refer to 1:2 oscillations and in the lower row 1:3 oscillations. In panels (a) and (e) we show the results from Monte Carlo method with fixed parameters and in (b) and (f) the points obtained when drawing the parameter values. Similarly, we compare the maps of the basin stability for fixed parameters (c,g) and obtained with the assumed mismatch (d,h). Results that refer to the remaining 7 solutions are presented in the supplement material.

The difference in the results from the extended basin stability analysis are barely visible despite the fact, that the positions of the bifurcation lines change when we modify the parameter values within the assumed ranges. This shows that the sample-based approach can be applied even without time consuming detailed measurements of the system’s parameters and still ensures sensible results. Instead of precisely measure the parameter values, we only have to asses the precision for each of the determined parameter.

## Conclusions

In this paper we present a critical comparison between the results obtained using the extended basin stability method, the path-following bifurcation analysis and experimental data. We investigate the double pendulum system which is a model of an existing experimental rig. It is a multistable system in which 9 different periodic solutions have been detected.

For each solution we use the path-following method to determine its range of stability in the two parameter space, namely the amplitude and the frequency of excitation. Then, we perform the experimental investigations for the same purpose. The obtained two parameter bifurcation diagrams allow us two determine ranges of stability and detect bifurcations that lead to destabilization of the investigated orbits. Finally, we apply the extended basin stability analysis to determine the ranges of stability and two parameters basin stability maps. Our results from both numerical methods are in a remarkably good agreement with the experimental data.

In comparison to classical methods the advantage of the presented method is that it enables us to analyse the influence of several parameters simultaneously. The computational effort does not increase significantly with the dimensions of the system. Moreover, the method enables us to detect hidden attractors and solutions with rather meagre basins of attraction. The method only requires an efficient numerical integration algorithm. There is no need of specific knowledge or software to use this method. The usefulness of a sample-base method is especially visible in higher dimensional systems where the classical analysis requires a preliminary study to know the initial conditions, the shape of periodic solutions, the proper cross-sections of the phase space, while the extended basin stability method can be applied straightforward without such specific knowledge.

Another advantage of the presented approach is that the collected data allow to quantify the stability of each attractor and prepare the two parameter basin stability maps. Such diagrams enable to detect the ranges in parameter space for which the solution is more likely to appear due to a large volume of the basin of attraction and have strong practical significance.

In the last part of the work we repeat the basin stability analysis under uncertainties in the parameter values. The obtained results do not differ significantly from the original data, hence we claim that the proposed method can be applied even without the precise knowledge of the parameters values which is a unique feature.

The above advantages predispose the sample-based approach for the analysis of multistable systems with large phase space dimensions and multiple co-existing attractors. Future application perspectives include especially analysis of large networks like power grids or simulations of brain dynamics.

The presented results are the first broad comparison between the path-following method, the basin stability approach and the experimental investigation. We show that the sample-based methods are a reliable tool for the analysis of complex dynamical systems. Moreover, we prove that the extended basin stability method has significant advantages which make it robust and appropriate for many applications in which classical analysis methods are difficult to apply.

## Electronic supplementary material

Supplementary Info
