# Erratum: Sample-based approach can outperform the classical dynamical analysis - experimental confirmation of the basin stability method

**DOI:** 10.1038/s41598-017-12315-5

**Published:** 2017-10-09

**Authors:** P. Brzeski, J. Wojewoda, T. Kapitaniak, J. Kurths, P. Perlikowski

**Affiliations:** 1 Division of Dynamics, Lodz University of Technology, 90-924 Lodz, Poland; 2 Potsdam Institute for Climate Impact Research, Potsdam, 14415 Germany; 3 Institute of Physics, Humboldt University of Berlin, Berlin, 12489 Germany

*Scientific Reports* **7**:6121; doi:10.1038/s41598-017-05015-7; Article published online 21 July 2017

The original version of this Article contained a typographical error in the spelling of the author T. Kapitaniak, which was incorrectly given as T. Kapitaniakenglish. This has now been corrected in the PDF and HTML versions of the Article.

