# Supplementary material for: Sample-based approach can outperform the classical dynamical analysis - experimental confirmation of the basin stability method
Source: Sci Rep. 2017 Jul 21;7:6121. doi: 10.1038/s41598-017-05015-7 (PMC5522450; doi:10.1038/s41598-017-05015-7)
Supplement: Supplementary file 1 — Supplementary Info [file 41598_2017_5015_MOESM1_ESM.pdf]

Supplementary material

Sample-based approach can outperform the classical dynamical analysis - experimental confirmation of the basin stability method

P. Brzeski[1,2,3,], J. Wojewoda[1], T. Kapitaniak[1], J. Kurths[2,3], P. Perlikowski[1]*

[1] *Division of Dynamics, Lodz University of Technology, 90-924 Lodz, Poland*

[2] *Potsdam Institute for Climate Impact Research, Potsdam 14415, Germany*

[3] *Institute of Physics, Humboldt University of Berlin, Berlin 12489, Germany*

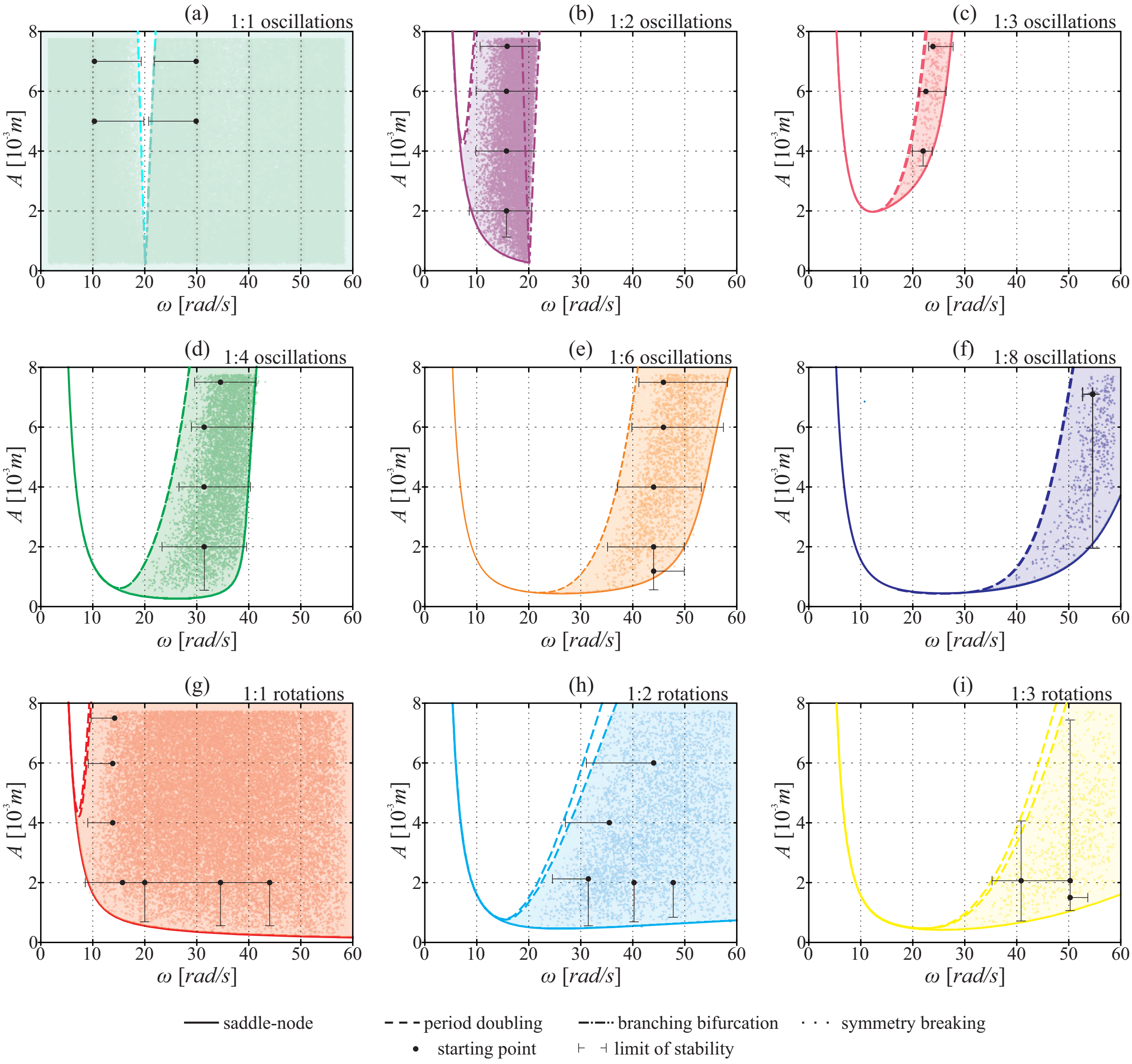

Figure 1: Results from the investigation using the extended basin stability method with parameters mismatch as described in Section "Investigation of the system with parameters mismatch". Figure corresponds to Fig. 3 that was obtained with fixed parameters values. Panels show the comparison between the ranges of stability obtained using the path-following method (colour lines), the sample-based approach (dots) and the experimental investigation (black lines). Each panel corresponds to a different solution.

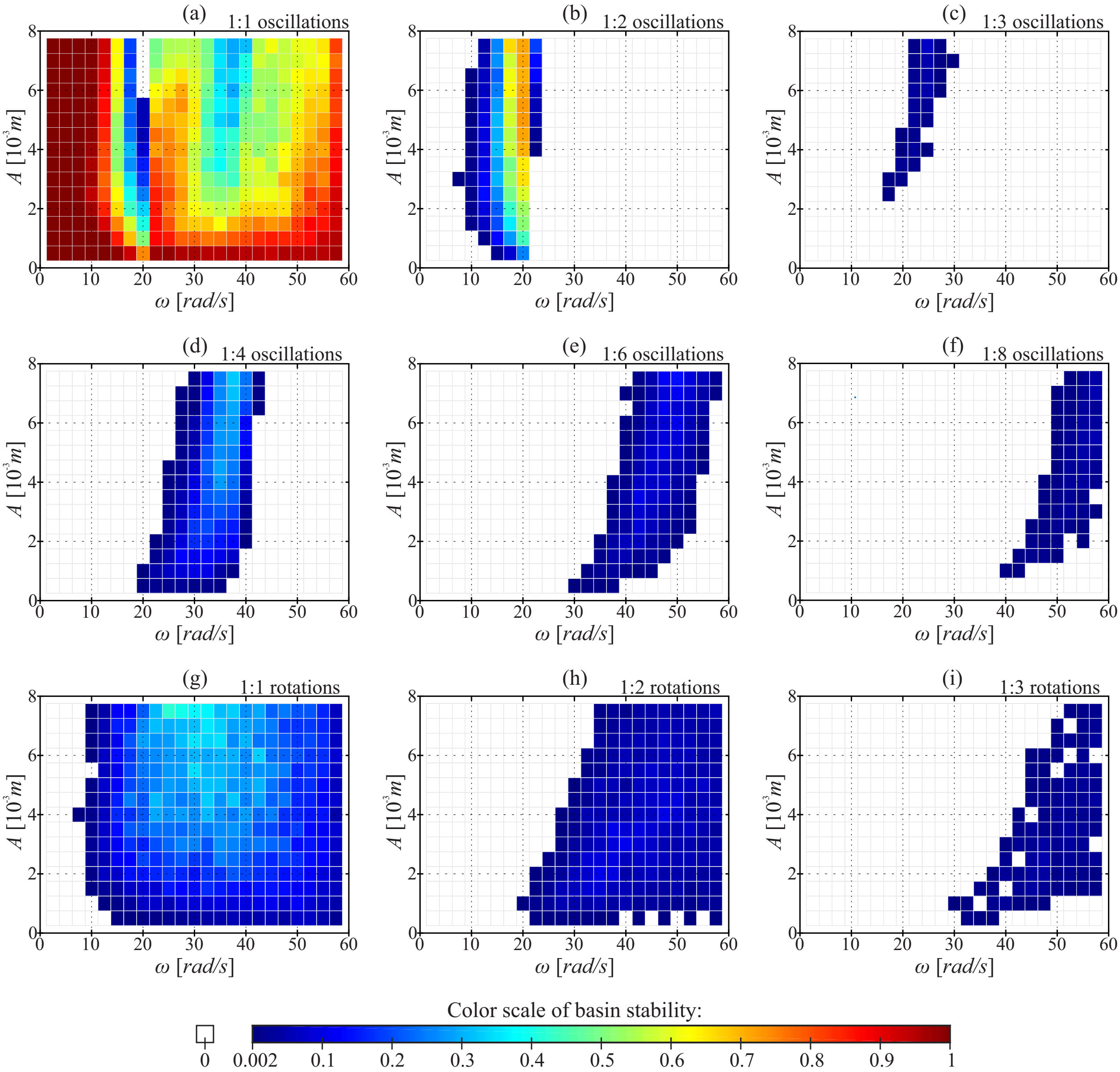

Figure 2: Results from the investigation using the extended basin stability method with parameters mismatch described in Section "Investigation of the system with parameters mismatch". Figure corresponds to Fig. 4 that was obtained with fixed parameters vaules. Panels show the two parameter maps of the basin stability for 9 investigated solutions. The grid corresponds to the lattice used during the calculations. Below is the common basin stability scale used for all 9 panels.
